# Developing and Evaluating the Comprehensive Hierarchical Eustress Review (CHER)

**DOI:** 10.1007/s10902-025-00999-w

**Published:** 2026-01-31

**Authors:** Juliane Kloidt, Lawrence W. Barsalou

**Affiliations:** School of Psychology and Neuroscience Glasgow, University of Glasgow, 62 Hillhead Street, Glasgow, G12 8QB UK

**Keywords:** Eustress, Stress, Wellbeing, Thriving, Challenges, Bifactor modelling

## Abstract

Psychometric research on eustress—the positive experience of a challenging situation—faces a variety of issues that include: What features of eustress are central to experiencing challenging situations positively? What psychometric structure best fits these features (unidimensional, multidimensional, bifactor)? How is eustress related to distress and wellbeing? Can individuals be clustered effectively into different eustress profiles? To address these issues, we developed a novel eustress instrument: the Comprehensive Hierarchical Eustress Review (CHER), motivated by a new model, the Comprehensive Hierarchical construct of Eustress (CHE). Analogous to the CHE model, the CHER instrument contains 3 subscales for CHE’s 3 sources of eustress (goal-directed action, momentary experience, stable qualities) with 47 items that reflect the 47 features of eustress that CHE extracted from the literature. To evaluate CHER and explore its potential for understanding eustress, we assessed it in a well-powered adult UK sample (*N* = 260). Using confirmatory factor analyses, we found that eustress is best understood as both a unidimensional and a multidimensional construct (i.e., a bifactor model), with items from all three subscales contributing to its conceptual core. The best performing model exhibited desirable internal qualities (satisfactory reliability and item discrimination) and external qualities (eustress negatively related to distress and positively related to wellbeing). Using latent profile analysis, we identified four clusters of individuals with different eustress profiles, who differed further on sociodemographic characteristics and personality traits. Findings reported have theoretical, empirical, and interventional implications for future work on the generation of positive experiences in challenging situations.

## Introduction

Negative experiences often occur when people experience difficult situations across diverse domains of human experience, including relationships, work, health, and finances. As research has documented for decades, negative stress experiences can have detrimental effects on physical and mental health, especially when these experiences occur chronically (O’Connor et al., 2021; Schneiderman et al., 2005). Notably, however, positive responses to difficult situations can also occur regularly, as when individuals experience accomplishment, effective coping, self-efficacy, and savoring the moment (Epel, 2020).

The distinction between negative and positive stress dates back to Selye’s model of Stress-as-Adaptation-Syndrome, characterizing physiological maladaptation to threatening situations as distress and physiological adaptation to challenging situations as eustress (Selye, 1936, 1976). The Transactional Model of Stress extended Selye’s distress-eustress distinction to include cognitive processes (Lazarus, 1966; Lazarus & Folkman, 1984, 1987). Whereas appraising a difficult situation as exceeding available coping resources can induce distress, appraising a challenge as falling within available resources can induce eustress.

Since these initial conceptualizations of distress and eustress, extensive research has established features that contribute to experiencing a challenging situation *negatively*, including expectation violation, perceived threat, and rumination (e.g., Lebois et al., 2016). It remains less clear, however, what features contribute to experiencing challenging situations *positively*. As a consequence, the term “eustress” has remained relatively vague and poorly understood, highlighting the need for more comprehensive theoretical models and measurement instruments (Kloidt & Barsalou, 2024; Le Fevre et al., 2003).

### Theories of Eustress

Diverse accounts of eustress, along with related evidence, can be found across relevant disciplines, including health psychology, occupational psychology, and educational psychology, and across diverse literatures, including those for basic, clinical, and applied research. Typically, each of these accounts conceptualizes eustress within the context of a specific project and/or research area, contributing useful yet relatively narrow and fragmented understandings of eustress. Comprehensive research on eustress that synthesizes insights and evidence across disciplines and literatures is in its infancy.

**Fig. 1:**
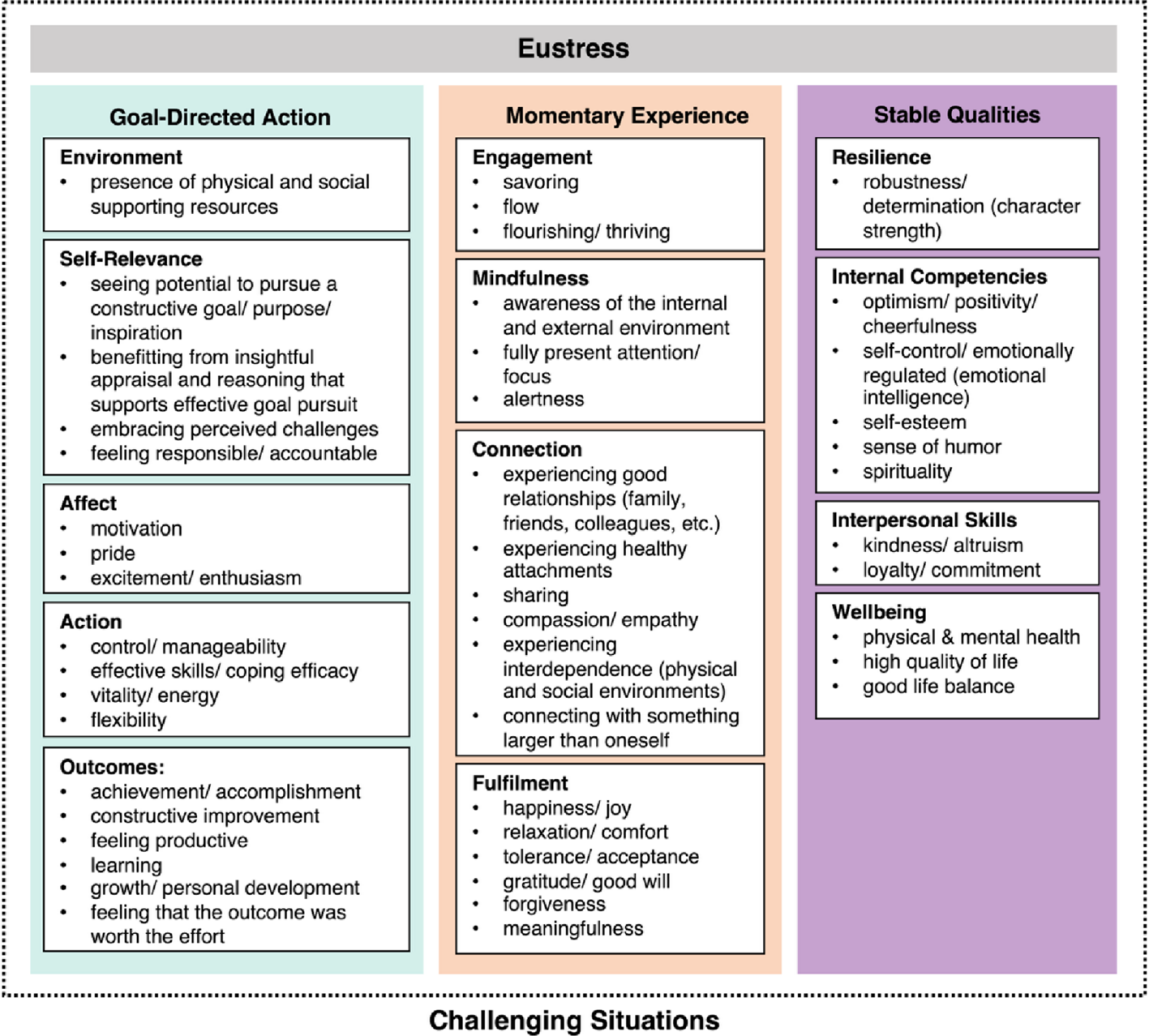
*C*omprehensive *H*ierarchical construct of *E*ustress (CHE). *Note.* At the highest level of organization, three eustress sources are depicted as colored columns (with bolded column headers). Together, the 3 sources contain a total of 13 mid-level facets, illustrated as white boxes within each column (bolded box headers). Finally, 47 low-level features of eustress are organized into the associated facet boxes (bullet points). Reproduced with permission from Kloidt and Barsalou (2024)

The first theoretical model of eustress to integrate cross-disciplinary research comes from Kloidt and Barsalou (2024), who identified 57 unique features of eustress through reviewing 80 theoretical, interventional, empirical, and psychometric articles. Using a deductive approach to theory-building, Kloidt and Barsalou structured the 57 identified features into a three-level hierarchical structure, the *C*omprehensive *H*ierarchical construct of *E*ustress (CHE). Figure 1 presents the CHE model (reproduced from Kloidt & Barsalou, 2024).

At CHE’s highest hierarchical level, eustress emerges from three general sources: goal-directed action, momentary experience, and stable qualities of the individual. At CHE’s intermediate level, the 3 general sources differentiate into 13 facets of eustress, each containing 1 to 6 of the original 57 features extracted from the eustress literature. For 10 of the 13 facets, its most representative feature was used to label the facet; for the other 3 facets, a new feature was induced to serve as its label. As Kloidt and Barsalou (2024) discuss, the facet labels are closely related to relevant theoretical models. Finally, at CHE’s lowest level, the remaining 47 features are nested within the 13 facets. As Fig. 1 illustrates, CHE’s sources, facets, and features capture the diverse forms that eustress states and traits can take across individuals and situations, covering the construct of eustress comprehensively.

According to CHE, one way eustress often occurs in challenging situations is from pursuing goal-directed action successfully, experienced, for instance, as insightful appraisal, effective coping, enthusiasm, and/or productivity. Eustress also results when individuals engage with challenging situations positively in the moment, experienced, for instance, as flow, mindfulness, social connection, and/or fulfilment. Lastly, individuals can develop stable qualities that support generating states of eustress in the moment, thereby making it possible to experience challenging situations positively instead of negatively. Stable qualities that enhance eustress include resilience, self-control, optimism, physical health, and mental health. Importantly, CHE focuses exclusively on generating positive experiences in *challenging* situations. As a consequence, the construct of eustress remains clearly differentiated from wellbeing, which typically occurs in *non-challenging* situations (although these constructs are also related; Kloidt & Barsalou, 2024).

### Psychometric Instruments for Assessing Eustress

Similar to theories of eustress, the development of psychometric instruments for assessing eustress in individuals remains at the early stages. To date, few eustress instruments exist (Cavanaugh et al., 2000; O’Sullivan, 2011; Rodríguez et al., 2013; Vikoler et al., 2024). Those that do only assess a fraction of the features of eustress established in CHE, thereby only offering partial coverage of the full construct. Existing psychometric instruments are further limited by assessing eustress in specific populations (e.g., students, managers, social workers) and/or in specific contexts (e.g., workplace settings including universities, corporations, or social services). None of the existing measures offers a comprehensive assessment of eustress in the general population across the diverse forms of eustress experience that CHE established. For these reasons, developing a psychometric instrument that assesses eustress comprehensively would be of value, not only for assessing individual differences in psychometric settings, but also in clinical, applied, behavior change, and research settings. Our primary goal here was to develop such an instrument.

### The Psychometric Structure of Eustress

Investigations into the psychometric structure of a construct, such as eustress, typically aim to answer questions about its central features and their factor structure. To measure eustress comprehensively, we used CHE’s 47 low-level features to develop 47 analogous items in a psychometric instrument. Because these 47 items capture the diverse forms that eustress takes across populations, contexts, and literatures, they can comprehensively measure an individual’s level of eustress. We didn’t include CHE’s facets and sources as candidate items because the 47 low-level features capture them all well. More importantly, including the facets and sources as test items would have produced unwanted and problematic redundancy in the resulting instrument. One central issue that we investigated in the study to follow is whether all 47 candidate items are relevant to the construct of eustress. Perhaps a subset of these features constitutes a conceptual core, with other features being peripheral (or redundant).

It also remains unclear how the 47 candidate items relate to one another and to the construct space of eustress that CHE established. One possibility is that eustress is a unidimensional construct. Traditional definitions of eustress, for example, often appear to suggest implicitly that eustress is a unidimensional construct, with all features contributing directly and similarly strongly (Lazarus & Folkman, 1984; Selye, 1974). Alternatively, eustress could be a multidimensional construct. Kloidt and Barsalou’s (2024) CHE model, for example, proposes that eustress emerges from three distinct sources, thereby proposing explicitly that it is a multidimensional construct. Interestingly, these two views need not be mutually exclusive. In a bifactor structure, the eustress construct could contain both a general eustress factor and specific multidimensional factors (cf. general vs. domain-specific intelligence; Deary, 2012). Evaluating our novel instrument allowed us to assess these possibilities.

### Relating Eustress to Distress and Wellbeing

Investigating the psychometric structure of a construct typically begins with internal evaluation of it, identifying the best-fitting model, establishing its robustness and assessing its reliability. It is also, essential, however, to evaluate the construct externally, assessing its relations with related constructs. For external evaluation, eustress has been assessed most prominently in relation to distress, with eustress and distress viewed as opposite poles of the same construct (e.g., Cohen et al., 1983) or as distinct divergent constructs (e.g., Rodríguez et al., 2013). An important question is whether an individual can only experience high distress or eustress in a situation (being highly correlated negatively) or can instead experience high levels of both (being weakly correlated or uncorrelated). The study reported here addressed this issue.

As a second external relation, we also assessed the relation between eustress and wellbeing. As Kloidt and Barsalou (2024) review, trait-level features of eustress and psychological wellbeing overlap conceptually, suggesting that these two constructs should be positively correlated across individuals. These two constructs, however, differ in an interesting way. Whereas eustress focuses exclusively on positive experience in *challenging situations*, wellbeing focuses on positive experience in *non-challenging situations*. Because of this difference, eustress and wellbeing may not be highly related. The study reported here allowed us to evaluate these possibilities.

### Eustress Profiles

Establishing the key features and structure of a comprehensive eustress construct informs understanding the basic processes that underlie it, while further supporting interventions to increase eustress in clinical, applied, behavior change, and research settings. Although existing research suggests that eustress can be a highly unique, individual experience (O’Sullivan, 2011; Rodríguez et al., 2013), it is likely that groups of individuals share eustress experiences to some extent. For this reason, it would also be useful if a eustress assessment instrument could also establish profiles of reliable differences at the group level, where different individuals exhibit similar eustress profiles.

The possibility that groups of individuals exhibit different eustress profiles follows from a person-centered approach to positive emotions, suggesting that individuals can be broadly categorized into “flourishers” and “languishers” depending on their capacity to engage positively with the moment and build resilience, personal resources, and wellbeing over time (cf. broaden-and-build theory; Fredrickson & Joiner, 2002; Keyes, 2002). Although it can also be useful to establish an idiosyncratic profile of eustress for each individual, establishing group profiles can offer insights into understanding general processes that contribute to a construct (including shared genes, environments, and/or epigenetic history), support generalizations across individuals, and guide precision medicine interventions for specific kinds of individuals.

### Study Overview

The current work aimed to address the theoretical and applied issues just presented. To do so, we developed a novel psychometric instrument that assesses eustress in the general UK population across challenging situations (i.e., not in specific populations or contexts). The Comprehensive Hierarchical Eustress Review (CHER) developed here contains 47 items that reflect CHE’s 47 low-level features (Fig. 1). These 47 items were further organized into 3 subscales reflecting CHE’s 3 multidimensional sources of eustress. To evaluate CHER, we assessed it in a well-powered sample of UK adults, together with psychometric instruments that measure related constructs.

Three research questions (RQs) were of primary interest:

**RQ1.** What psychometric model best describes eustress as measured by CHER? Unidimensional, multidimensional, or both (bifactor)?

**RQ2**. Does CHER’s best performing model exhibit desirable internal and external psychometric qualities?

**RQ3.** Can CHER’s best performing model be used to organize individuals into distinct clusters that reflect distinct group-level profiles of eustress?

To address RQ1, we first investigated how to best model the factor structure that underlies eustress, using confirmatory factor analysis on the dataset collected with CHER here. If eustress is best understood as a simple unidimensional construct, then a one-factor model should offer an excellent fit to the 47 features of eustress that CHER assesses. Alternatively, the structure of eustress could reflect CHE’s three multidimensional sources: goal-directed action, momentary experience, and stable qualities. If so, then a three-factor model should offer an excellent fit of the data collected with CHER. Still another possibility is that eustress reflects both a general eustress factor, accompanied by specific multidimensional factors. If so, then a bifactor model should fit the data collected with CHER well.

To address RQ2, we evaluated the best-fitting model from RQ1 internally and externally. For internal evaluation, we established CHER’s test reliability and item discriminatory power. For external evaluation, we assessed the relationship between CHER’s measure of individual eustress with another measure of eustress, and also with measures for distress and wellbeing.

To address RQ3, we used latent profile analysis to identify different groups of individuals who shared differed eustress profiles on CHER. Specifically, we established groups who exhibited similar patterns across factor scores from the best-fitting model in RQ1. We then investigated whether belonging to a specific cluster was systematically associated with sociodemographic characteristics, personality traits, distress, and wellbeing.

## Methods

### Participants

This study received ethics approval (please see Ethical Approval section). We recruited a gender-balanced, non-probabilistic sample through the Prolific online platform that met the following inclusion criteria: UK residents, aged 18 to 80 years old, self-reported English fluency, and completion of at least 20 Prolific studies with a 100% approval rate. Specifically, we recruited 260 participants to meet minimum requirements for stable correlations (*n* > 250; Schönbrodt & Perugini, 2013) and to ensure convergent solutions for confirmatory factor models (*n* > 100; MacCallum et al., 1999) and bifactor models (*n* > 150; Bader et al., 2022). We inspected our data for mechanical or random responses but refrained from excluding outliers, as we expected highly variable distributions reflecting large individual differences.

All participants passed data quality checks that checked for random and mechanical responding, resulting in a final sample of 260 UK adults (128 male, 127 female, 3 genderqueer/non-binary, 2 prefer not to say) with a mean age of 44.60 years (*SD* = 13.60). Participants spent a mean time of 15 years (*SD* = 3.03) in formal education and most commonly reported that their annual income ranged from £20,001 and £30,000 (*n* = 69), with both measures approximating UK population levels (Office for National Statistics, 2021, 2025).

### Materials

#### Comprehensive Hierarchical Eustress Review (CHER)

The CHER psychometric instrument contains 47 self-report items that closely reflect the 47 low-level eustress features of the Comprehensive Hierarchical construct of Eustress (CHE) illustrated in Fig. 1. As described earlier, these features were derived from a scoping review of the eustress literatures (Kloidt & Barsalou, 2024). Table 1 presents CHER’s 47 test items.

Following CHE’s hierarchical structure, we grouped similar test items into distinct facets and then organized facets into three subscales reflecting CHE’s general sources of eustress: goal-directed action (18 items), momentary experience (18 items), and stable qualities of the individual (11 items). As Table 1 illustrates, we integrated subscale affiliations into the wording of the survey items, given that doing so was necessary for conveying the content of each item accurately. Items were evaluated on a continuous self-report scale with one decimal point precision, ranging from 0 (not at all) to 10 (a lot).

**Table 1:**
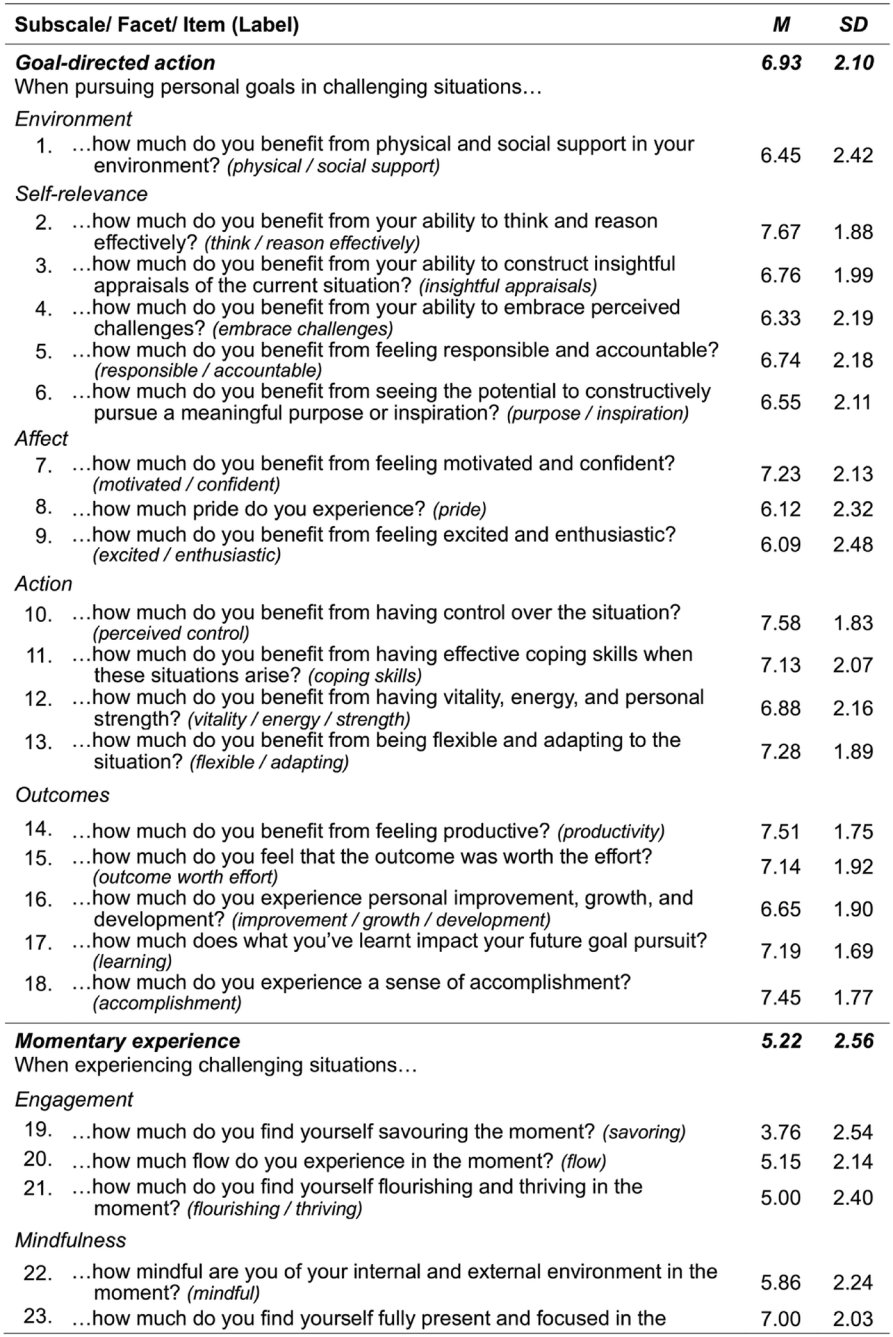

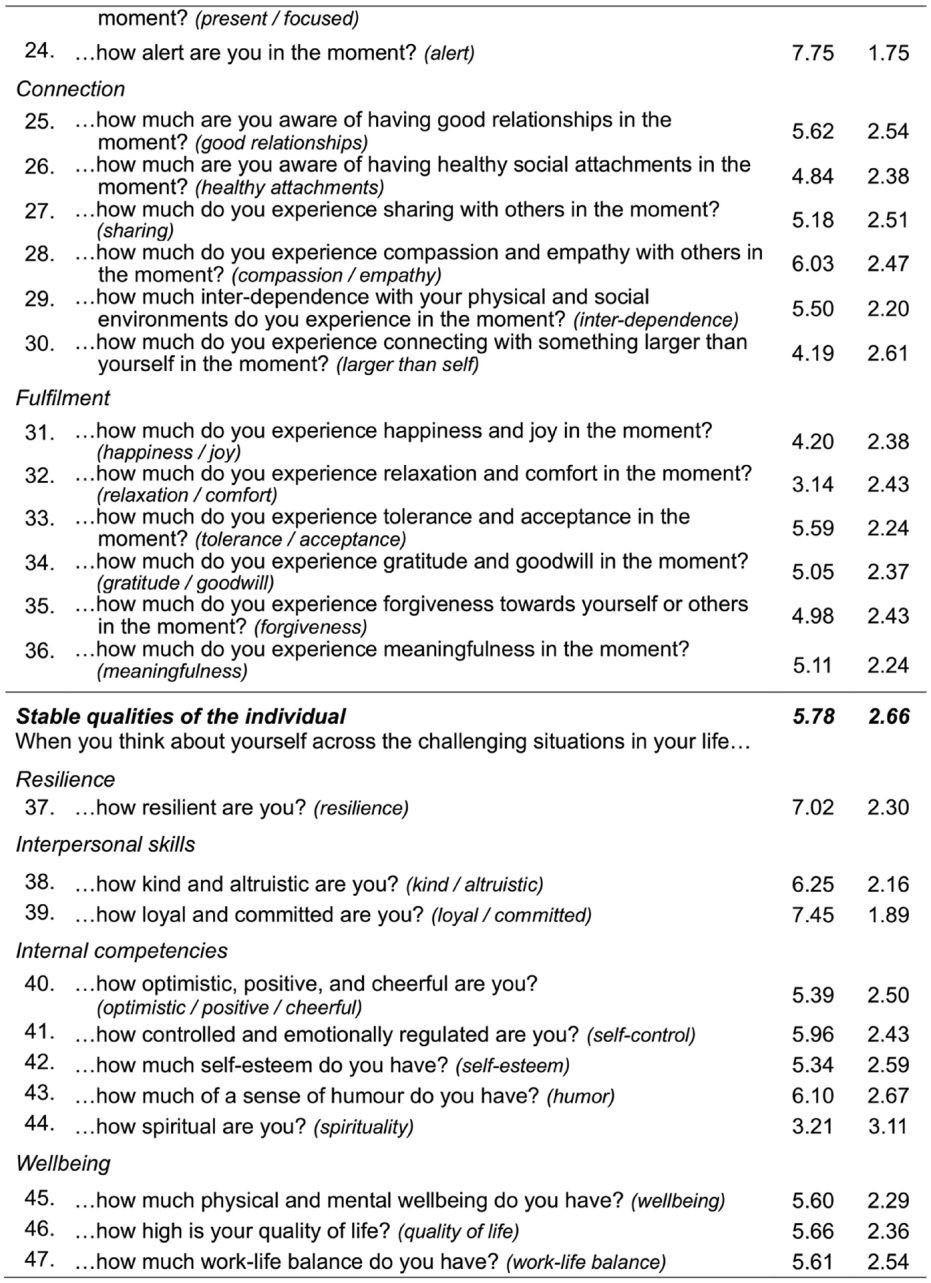
The 47 test items in the Comprehensive Hierarchical Eustress Review (CHER), clustered by source and facet

*Note*. Participants rated items on continuous 0 to 10 self-report scales (one decimal place precision) with lower scores indicating lower levels of eustress. Scale labels were “not at all” (0), “moderately” (5), and “a lot” (10). To better fit the wording of the items for *pride, self-esteem, humor, wellbeing,* and *work-life balance*, the labels used were “none” (0), “moderately” (5), and “a lot” (10); similarly, the item for *quality of life*, used the labels “very low” (0), “moderate” (5), and “very high” (10). Please refer to SM-1 for additional item-level descriptives. *M* = mean; *SD* = standard deviation. *N* = 260

#### Perceived Stress Scale (PSS-10; Cohen et al., 1983)

The 10-item PSS measures how much distress individuals experienced during the last month. Items were evaluated on a continuous self-report scale with one decimal point precision, ranging from 0 (never) to 4 (very often). Across several studies, the PSS-10 has exhibited satisfactory test reliability (*α* = 0.74 − 0.91) and test-retest reliability (*r* >0.70; Lee, 2012). In our sample here, the PSS-10 exhibited excellent test reliability (*α* = 0.94).

#### Perceived Eustress Scale (PES-10)

The PES is an unpublished instrument that was developed and evaluated in our research group (presented in SM-2). Designed to have analogous structure and content in relation to the PSS, the 10-item PES assesses how much eustress individuals experienced during the past month. Items were evaluated on a continuous self-report scale with one decimal point precision, ranging from 0 (never) to 4 (very often). In a three-timepoint study over two weeks, the PES-10 exhibited satisfactory test reliability (α = 0.84) and adequate test-retest reliability that appeared to also reflect systematic change in eustress across the two-week period (*r* =0.63 −0.68). In our sample here, the PES-10 exhibited a similarly satisfactory test reliability (*α* = 0.85).

#### European Social Survey Wellbeing Module (ESS Wellbeing; Huppert & So, 2013)

The 10-item ESS Wellbeing instrument assesses 10 dimensions of psychological wellbeing that were evaluated in a representative sample of 43,000 Europeans from 23 countries (Huppert & So, 2013). Items were evaluated on continuous self-report scales here with one decimal point precision. To account for differing response scales and item weights, we computed standardized factor scores with higher scores representing increased wellbeing (Ruggeri et al., 2020). In our sample here, the ESS Wellbeing exhibited satisfactory test reliability (*α* = 0.83).

#### HEXACO-60 (Ashton & Lee, 2009)

The 60-item HEXACO instrument assesses personality traits along the six dimensions of honesty, emotionality, extraversion, agreeableness, conscientiousness, and openness. Items were evaluated here on a continuous self-report scale with one decimal point precision, ranging from 1 (strongly disagree) to 5 (strongly agree). All subscales have shown satisfactory test reliability (α = 0.73 − 0.80) and test-retest reliability (*r* = 0.82 − 0.89) in a large adult sample (Henry et al., 2022). In our sample here, the test reliabilities of the HEXACO subscales ranged from from 0.79 (honesty) to 0.87 (extraversion).

#### Socioeconomic Characteristics

We collected socioeconomic information that included time spent in formal education (ranging from 0 to 20 + years) and annual income (divided into £10,000 increments ranging from “below £10,000” to “above £50,000”). We further measured subjective social status with the MacArthur Scale (Adler et al., 2000), where participants placed themselves on a 10-rung ladder representing where people stand in the United Kingdom (with higher rungs indicating higher subjective social status). This measure has exhibited adequate test-retest reliability in a large multi-ethnic sample (*r* = 0.62; Operario et al., 2004).

### Procedure

Following recruitment via Prolific, participants were referred to the Qualtrics platform, where they provided informed consent and completed the survey online. In a within-group design, all participants completed the CHER scale, followed by the HEXACO-60, the PSS-10, the PES-10, the ESS Wellbeing, and sociodemographic questions. For each individual, the 47 CHER items were presented in a random order to break up items from the same subscale. Items in all other psychometric instruments were presented in a fixed order, as typically followed when implementing them. After study completion (*Mdn* = 20 min, *IQR* = 10 – 29 min), participants were debriefed, redirected back to Prolific, and compensated with £2.50.

### Data Analysis

We performed statistical analyses in R (v4.3.2; R Core Team, 2023) and RStudio (v2023.12.1 + 402; RStudio Team, 2022), with the packages tidyverse (Wickham et al., 2019), psych (Revelle, 2023), lavaan (Rosseel, 2012), and tidyLPA (Rosenberg et al., 2018). Data and code are available on OSF (https://osf.io/s7mgy/).

## Results

### RQ1: Modelling the Psychometric Structure of Eustress

To investigate the psychometric structure of eustress, we performed a series of confirmatory factor analyses. Specifically, we first assessed models with a single factor (implying that eustress is unidimensional) versus models with multiple correlated factors (implying that eustress is exclusively multidimensional). We then implemented a bifactor model to address whether the structure of eustress simultaneously reflects a general factor and three group factors for CHE’s three sources of eustress (Reise, 2012; Reise et al., 2010).

Because CHER is a novel instrument, we also examined whether all 47 items are essential for capturing the construct of eustress and removed poorly loading items from our initial factor solutions. To strike a reasonable balance between conservative item cut-offs (keeping the most conceptually important items only; Tabachnick & Fidell, 2007) versus liberal cut-offs (retaining a comprehensive sample pool of items; Stevens, 1992), we employed stepwise removal of items with main factor loadings < |0.50|. The chosen cut-off therefore only retained items for which the factor explained at least 25% of variance, a figure obtained through squaring its loading (cf. Brown, 2015).

To evaluate how well the CHER data fit each model’s predicted structure, we report the following fit indices: comparative fit index (CFI), Tucker-Lewis index (TLI), root mean squared error of approximation (RMSEA), Bayesian nformation criterion (BIC), and Akaike information criterion (AIC). For acceptable fit, CFI and TLI should be > 0.90 whereas RMSEA should be < 0.08 (Brown, 2015). Lower relative values for the BIC and AIC indicate more parsimonious solutions. Factor loading tables for all initial models with all 47 items are available in the Supplemental Materials (Tables SM-3, SM-4, SM-5).

#### Model 1 (unidimensional)

We first investigated whether a traditional definitional understanding can sufficiently cover the construct of eustress. Thus, this first model (M1) consisted of one general factor in a confirmatory factor analysis, assuming that eustress is a unidimensional construct without any specificity that reflects multidimensionality. Although all 47 items of the CHER instrument loaded positively on the eustress factor, 10 item loadings were below the enforced cut-off at 0.50 (again, all loadings can be found in SM-3). The stepwise removal of these items resulted in a factor solution that contained 37 items (Figure 5 in the Appendix). Their mean factor loading was 0.63, with the single latent eustress factor explaining 40.41% of the total item variance. Although the large mean factor loading indicates meaningful measurement of a general eustress construct, model fit was not acceptable according to our fit criteria, CFI = 0.723, TLI = 0.707, RMSEA (90% confidence interval) = 0.099 (0.095 − 0.104), with relative fit values BIC = 38,447 and AIC = 38,183. We therefore investigated whether a multidimensional model—as predicted by CHE—fits the structure of eustress better.

#### Model 2 (multidimensional)

The second model (M2) assessed CHE’s structure in Fig. 1, examining whether multiple factors for goal-directed action, momentary experience, and stable qualities capture the structure of eustress effectively. Specifically, M2 implemented a confirmatory factor analysis that assessed how these three CHER subscales fit the data. Items could only load onto their respective factor, but we permitted inter-factor correlations to capture potential overlap between M2 factors. After removing 7 items with factor loadings < 0.50, 17 items loaded on the goal-directed action factor, 15 on the momentary experience factor, and 8 on the stable qualities factor (see Figure 6 in the Appendix; all loadings can be found in SM-4).

Compared to M1, M2 retained more items and exhibited higher mean factor loadings of 0.68 for goal-directed action, 0.67 for momentary experience, and 0.70 for stable qualities. M2 explained 46.54% of total item variance (more than M1) and exhibited improved (yet not satisfactory) model fit for CFI = 0.829, TLI = 0.819, and RMSEA (90% *CI*) = 0.074 (0.069 − 0.078), but worse fit for BIC = 41,334, and AIC = 41,038. These results indicate that CHE’s theory-driven multidimensional structure captured CHER’s diverse item pool somewhat better than a unidimensional structure. Nevertheless, strong inter-factor correlations (*M* = 0.76, range = 0.67 − 0.81) suggested poor discriminant validity between the factors, implicating the presence of a general factor in the data set that complemented multidimensionality at the group-level (i.e., taking the form of specific factors for CHE’s three sources). To assess this possibility, we combined M1 and M2 into a bifactor model, assessed next.

#### Model 3 (bifactor)

Of primary interest here was whether a general eustress factor is present in the dataset, as the high correlations between the three M2 factors suggest. Because M1 ruled out a single factor model, this third model (M3) allowed for the general factor to be accompanied by group factors that represent CHE’s three multidimensional subscales for goal-directed action, momentary experience, and stable qualities. M3 therefore included the same three-factor structure as M2 but with an additional general eustress factor. All factors were orthogonal, ensuring high discriminant validity.

**Fig. 2:**
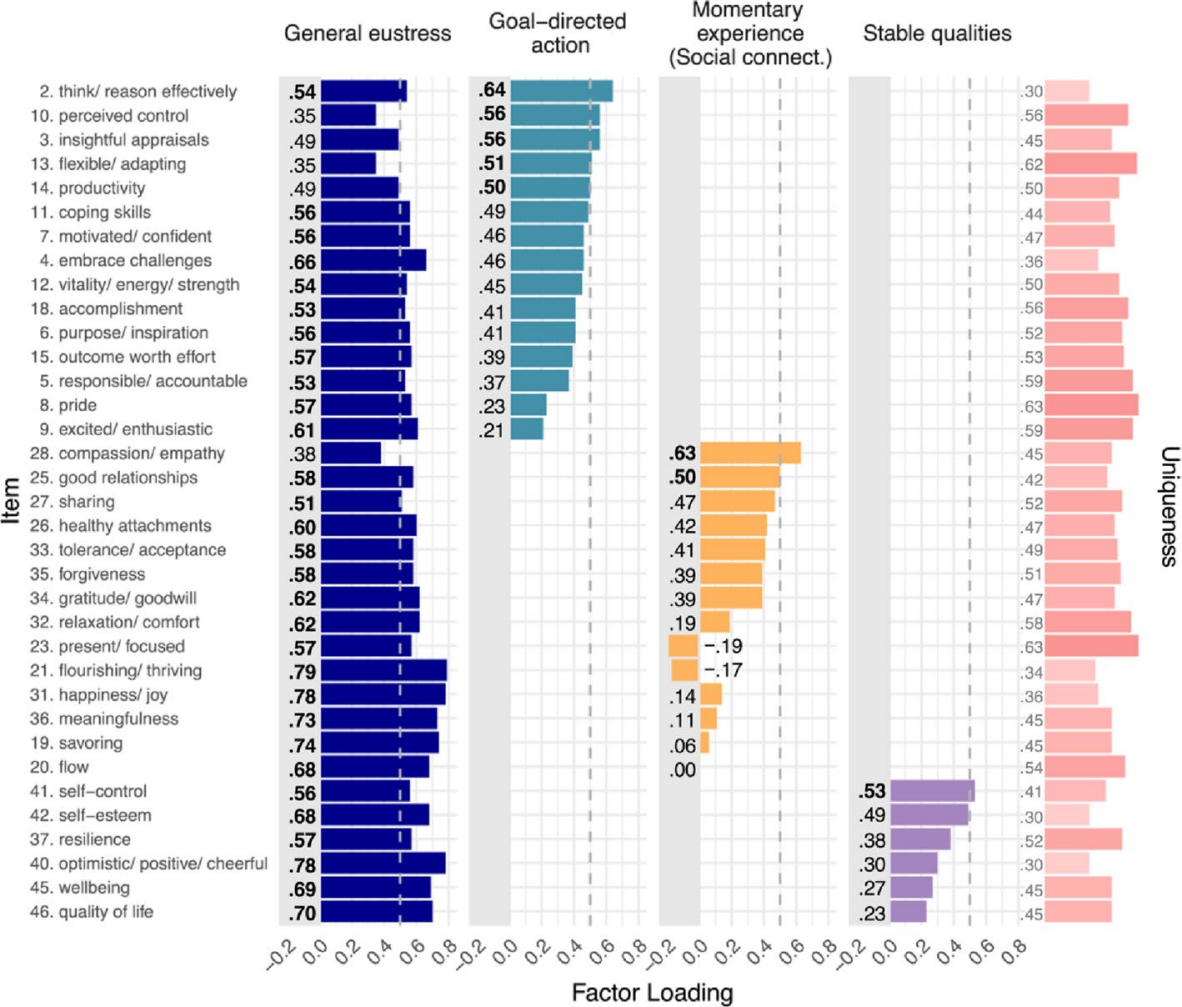
Factor loading plot for Model 3 (bifactor). *Note.* M3 captured 35 of CHER’s original 47 items, with main factor loadings for them ≥ |0.50| (dashed gray line). The general eustress factor included all 35 items, with the 3 group-level factors containing subsets of the 35 items, specifically, goal-directed action (15 items), momentary experience (14 items), stable qualities (6 items). M3 accounted for 52.17% of total variance, with most variance explained by general eustress (68.96%) followed by goal- directed action (17.15%), momentary experience (9.07%), and stable qualities (4.82%). Factor loadings ≥ |0.50| are bolded. Light gray rectangles distinguish negative factor loadings. Social connect. = Social connection. *N* = 260

Whereas the initial 47-item bifactor model had limited conceptual interpretability due to *negative* factor loadings for some items ≥ |0.50|, the stepwise removal of 12 weakly loading items < |0.50| resulted in a clearly interpretable solution with all meaningful loadings being positive (SM-5 presents all loadings). As Fig. 2 illustrates, the general eustress factor included all the remaining 35 items. Additionally, the group factor for goal-directed action included 15 items; the group factor for momentary experience included 14 items; and the group factor for stable qualities included 6 items. M3 accounted for 52.17% of total variance (more than M1 and M2) and close-to-acceptable model fit, CFI = 0.881, TLI = 0.865, RMSEA (90% *CI*) = 0.069 (0.064 − 0.075), with BIC = 35,966, and AIC = 35,592, lower than for either previous model.

The general eustress factor in M3 explained 68.96% of the extracted common variance and had a mean factor loading of 0.59, which was comparable to the mean factor loading in the unidimensional solution M1 (*λ*_*mean*_ = 0.63). The three group factors captured residual variance in their respective items after the general factor first accounted for common variance across all items. For goal-directed action and stable qualities, all their respective items exhibited meaningful loadings on the respective group-factor (*λ*_*mean*_ = 0.44 and 0.37, respectively). For momentary experience, items related to social connection exhibited meaningful loadings (*λ*_*mean*_ = 0.50), whereas loadings for the remaining items did not (*λ*_*mean*_ = 0.13). It therefore appears that the momentary experience factor in the bifactor model captured social connection, whereas all the remaining features associated with (non-social) momentary engagement were captured by the general eustress factor. M3 therefore suggests that the construct of eustress, as measured with CHER, is best represented by a general factor (emphasizing momentary engagement and stable qualities) and three group factors for goal-directed action, momentary experience (emphasizing social connection), and stable qualities.

#### Model Comparison and Selection

**Fig. 3:**
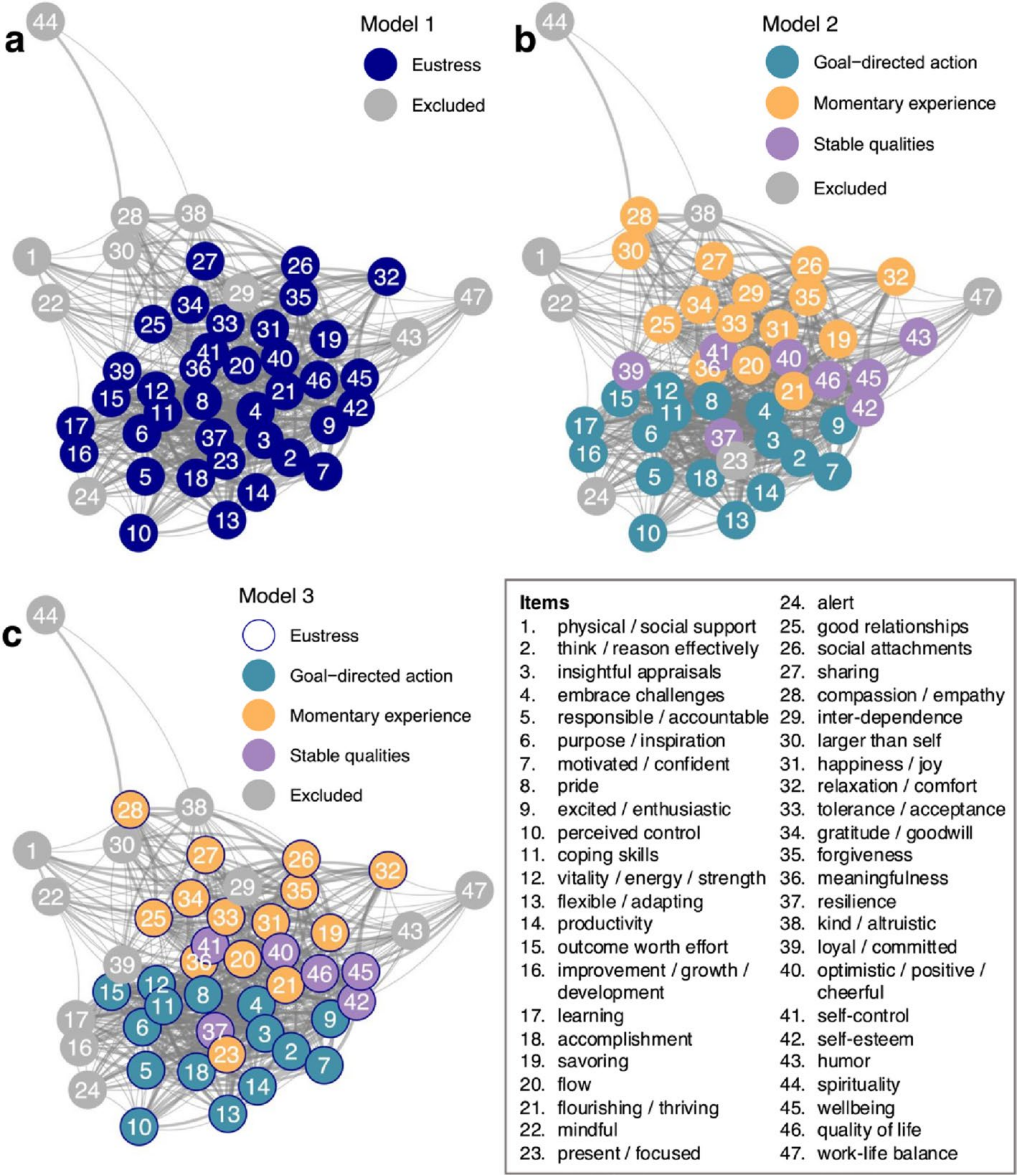
Network plots illustrating the construct space of eustress with different clustering solutions. *Note.* Nodes depict the 47 items of the CHER scale with node labels specifying corresponding CHER features. Same-colored nodes depict the factor(s) in Model 1 (**a**), Model 2 (**b**), and Model 3 (**c**). For Model 3 (**c**), dark blue node outlines indicate the general eustress factor in addition to group factors (coloured node fill). Gray nodes depict weakly-loading items that were excluded during model fine-tuning. Edges depict Spearman correlations between item pairs > 0.30 with wider edges indicating larger correlations. *N* = 260

To better understand the components and structure of the three models, we visualized the 2,162 pairwise correlations between the 47 CHER items in a network plot for each. Figure 3 presents the results, with colored nodes representing items included in each of the three confirmatory models, and with gray nodes representing excluded items (included here to further motivate their exclusion from the confirmatory models, but not included in any subsequent analyses). What is perhaps most striking across the three plots is that the included items form a single large cluster in the center of the network, with the excluded items dispersed around its periphery.

Across network plots, the highly inter-correlated core of the network essentially constitutes the general eustress factor in the M1 and M3 models. What is striking about this core, however, is that it contains well-segregated regions for CHE’s three multidimensional sources of eustress. Specifically, as the panels for M2 and M3 illustrate, properties associated with momentary experience reside at the top of the core cluster, properties associated with goal-directed action reside at the bottom, and properties associated with stable qualities reside on the boundary between these two clusters, especially on the right. In other words, a general core construct of eustress consists of three components, each constituting one of CHE’s three sources of eustress.

We next proceeded to formally establish the best-performing model for further analysis, striving for balance between reasonable model fit criteria and conceptual interpretability. Although M1 established that a general factor meaningfully explains shared variance across the measurement items, model fit was inadequate. From the additional perspectives of M2 and M3, M1’s poor fit reflects the fact that it doesn’t capture the multidimensional structure embedded within the core eustress construct (Fig. 3). We therefore dismissed M1, rejecting the assumption that eustress, as measured with the CHER scale, is exclusively unidimensional.

In comparison to M1, the correlated factors in M2 retained more items, explained more item variance, had better model fit (except for BIC and AIC values), and reflected the existing CHE model of eustress. Nevertheless, M2 exhibited strong inter-factor correlations, suggesting that a general factor does indeed underlie eustress, consistent with M1. Because M2 failed to capture this core factor, we therefore dismissed M2, together with the assumption that eustress, as measured with the CHER scale, is exclusively multidimensional.

Combining the general factor of M1 with the theory-based factors of M2, the bifactor model M3 explained the most total variance and exhibited the best model fit, including substantially lower values for BIC and AIC. We note that the model fit indices CFI and TLI were just slightly below acceptable cut-offs, possibly indicating unstable values that reflect our sample size (Garrido et al., 2016). M3 also provided a compelling factor structure. Specifically, all M3’s 37 included items loaded on the general factor for eustress. Additionally, all items associated with goal-directed action and stable qualities loaded meaningfully on their respective group factor. Interestingly, however, only half the items for momentary experience loaded meaningfully on its group factor, namely, the items related to social connection. All the other items *not* related to social connection were related to momentary engagement instead. Because the general factor explained most of the variance associated with these latter items, it follows that momentary engagement was central to the general eustress factor. Indeed, as Fig. 2 illustrates, the highest-loading items on the general eustress factor were those related to momentary engagement.

For M3, the general factor captured the highest proportion of explained item response variance (68.96%), again, with momentary engagement items from the momentary experience subscale being most important, together with items for stable qualities (Fig. 2). M3 further benefitted from group factors for goal-directed action, momentary experience (social connection), and stable qualities, accounting for an additional 17.15%, 9.07%, and 4.82% of the explained variance, respectively.

To ensure that M3 is robust under our sample size, we ran 500 Monte-Carlo simulations of our final bifactor solution post hoc. The results exhibited a highly satisfactory convergence rate of 100%, where the same factor structure presented above was observed. Additionally, estimates of the explained common variance for the general eustress factor on average exceeded population values by 0.001, corresponding to an acceptable relative bias of -0.001 (for a tutorial, see Bader et al., 2022). Based on the overall pattern of results for M3, we selected it as the best-performing model. We further concluded that eustress is primarily unidimensional and also somewhat multidimensional, with a core set of items robustly capturing a general eustress construct that is further partitioned into weaker components for CHE’s three sources of eustress (Fig. 3).

### RQ2: Evaluating the Best Performing Model Internally and Externally

Having selected M3 as the best-performing model, we next evaluated it internally (establishing its test reliability, the proportion of variance attributable to the general factor, and discriminatory power) and externally (comparing it with related instruments for distress, eustress, and wellbeing).

#### Internal Evaluation

We first calculated coefficient alpha (*α*) to establish the test reliability of M3’s factor scores (Shrout & Fleiss, 1979). Across factors, *α* was highly acceptable: 0.89 for stable qualities, 0.92 for momentary experience, 0.93 for goal-directed action, and 0.96 for general eustress. Because our measure was not designed to be tau-non-equivalent, we could not estimate its internal consistency with *α.* We instead calculated coefficient omega hierarchical (*ω*_*h*_) to investigate how well the total scores for M3 measured a saturated core construct of eustress across all factors (Flora, 2020; Reise et al., 2010). For M3, the proportion of total-score variance accounted for by the general factor (despite the multidimensional nature of the instrument) was 0.85, again exhibiting an acceptable value.

We next assessed the discriminatory power of the 35 CHER items in M3 using item-total correlations between each specific item and its corresponding factor score (excluding the respective item). Positive coefficients indicate that individuals with a high factor score are more likely to endorse the item, whereas coefficients close to zero or below zero indicate that the item discriminates poorly between individuals with high versus low factor scores. Table SM-6 shows that all item-total correlations with the M3 factor scores exhibited satisfactory discriminatory power, with mean correlations ranging from 0.62 (general eustress) to 0.71 (stable qualities). Three items (*flexible/adapting*, *perceived control*, *compassion/empathy*) had reduced item-total-correlations with the general factor, reflected in their peripheral network location (Fig. 3c). In addition, the item *present/focused* had a reduced item-total-correlation with its specific factor, again reflected by its distanced position to other items from the momentary experience subscale (Fig. 3c).

#### External Evaluation

To evaluate M3 externally, we assessed whether M3’s factors exhibited the expected relationships with related psychometric measures of distress, eustress, and wellbeing. Specifically, we correlated the factor scores for M3 with the Perceived Stress Scale (PSS-10; Cohen et al., 1983), the Perceived Eustress Scale (PES-10), and the ESS Wellbeing Module (ESS Wellbeing; Huppert & So, 2013). Table 2 illustrates that—in line with our predictions—the general eustress factor exhibited a significantly negative correlation with the PSS-10 and significantly positive correlations with the PES-10 and the ESS Wellbeing, indicating satisfactory discriminant and convergent qualities, respectively.

**Table 2:**
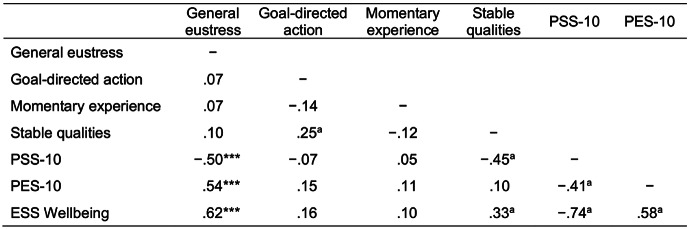
Model 3 (bifactor): Spearman correlations with related constructs

*Note*. Spearman correlations between participants’ standardized M3 factor scores, Perceived Stress Scale (PSS-10), Perceived Eustress Scale (PES-10), and the European Social Survey Wellbeing Module (ESS Wellbeing). *N* = 260. Significant correlations for one-sided confirmatory predictions are marked with * *p* <.05; ** *p* <.01; *** *p* <.001. Significant correlations for two-sided exploratory analyses with Bonferroni correction (*α* = 0.05/18) are marked with a *p* <.003. Unmarked correlations reflect non-significant exploratory analyses.

Notably, general eustress correlated most strongly with psychological wellbeing, indicating that individuals’ positive experiences may be similar in both challenging and non-challenging situations (see Kloidt & Barsalou, 2024, for relevant discussion). The moderate correlations of the PSS-10 with M3’s general eustress factor and with the PES-10 indicate that distress and eustress are not mirror images of each other but differ to some extent. The moderate correlation of the general eustress factor with the PES-10 probably reflects the fact that the PES-10 primarily contains items related to goal-directed action but not to momentary experience or stable qualities (see SM-2).

In contrast to the general eustress factor, exploratory analyses revealed few relations between group-factors and external measures. Stable qualities exhibited sizeable and significant correlations with the PSS-10 and the ESS Wellbeing in the same directions as the general eustress factor, potentially because the three measures assess related constructs at the trait level. In contrast, the group factors for goal-directed action and momentary experience didn’t exhibit any significant relationships with the other measures. These results are not surprising, given that group factor scores in a bifactor model represent residual variance between subgroups of items after first contributing to common variance for the general factor. As a result, an item’s residual variance may often be only weakly related or non-related to a group-level factor. Additionally for momentary experience, only half of the items contributed meaningful loadings on this group-level factor, whereas the other weakly loading items may have created noise. Modest correlations of the group factors may therefore be attributed to structural requirements of the underlying model rather than to limited conceptual importance.

### RQ3: Identifying Individuals who Exhibit Distinct Profiles of Eustress

To examine whether groups of individuals with distinct eustress profiles exist within our sample, we conducted a series of latent profile analyses (LPA). Specifically, we used individual factor scores from the best-performing model M3 (bifactor model) as indicator variables to cluster individuals who exhibit similar configurations of eustress factor scores. Once we established latent profiles, we assessed whether individuals across clusters differed systematically in terms of sociodemographic characteristics, personality traits, distress, and wellbeing. To do so, we entered variables of interest as predictors into a multinomial logistic regression model.

#### Distinct Profiles of Eustress

 Using maximum likelihood estimation, we initially estimated latent profile solutions ranging from one to six distinct clusters of individuals. Table 3 in the Appendix presents comparisons of these candidate solutions that consider convergence issues, model fit statistics (i.e., information criteria, likelihood-ratio tests, and Bayesian indices), and diagnostic criteria (i.e., class size, average latent class probability, and entropy). After dismissing the 5-cluster solution due to convergence issues, we identified the 4-cluster solution as the best fitting model according to most fit statistics, including the Bayesian information criterion (BIC), consistent Akaike information criterion (CAIC), and Bayes factor (BF). The 4-cluster solution presented with acceptable average latent class posterior probabilities (range = 0.684 − 0.916) and entropy (0.732), suggesting appropriately separated clusters.

**Fig. 4:**
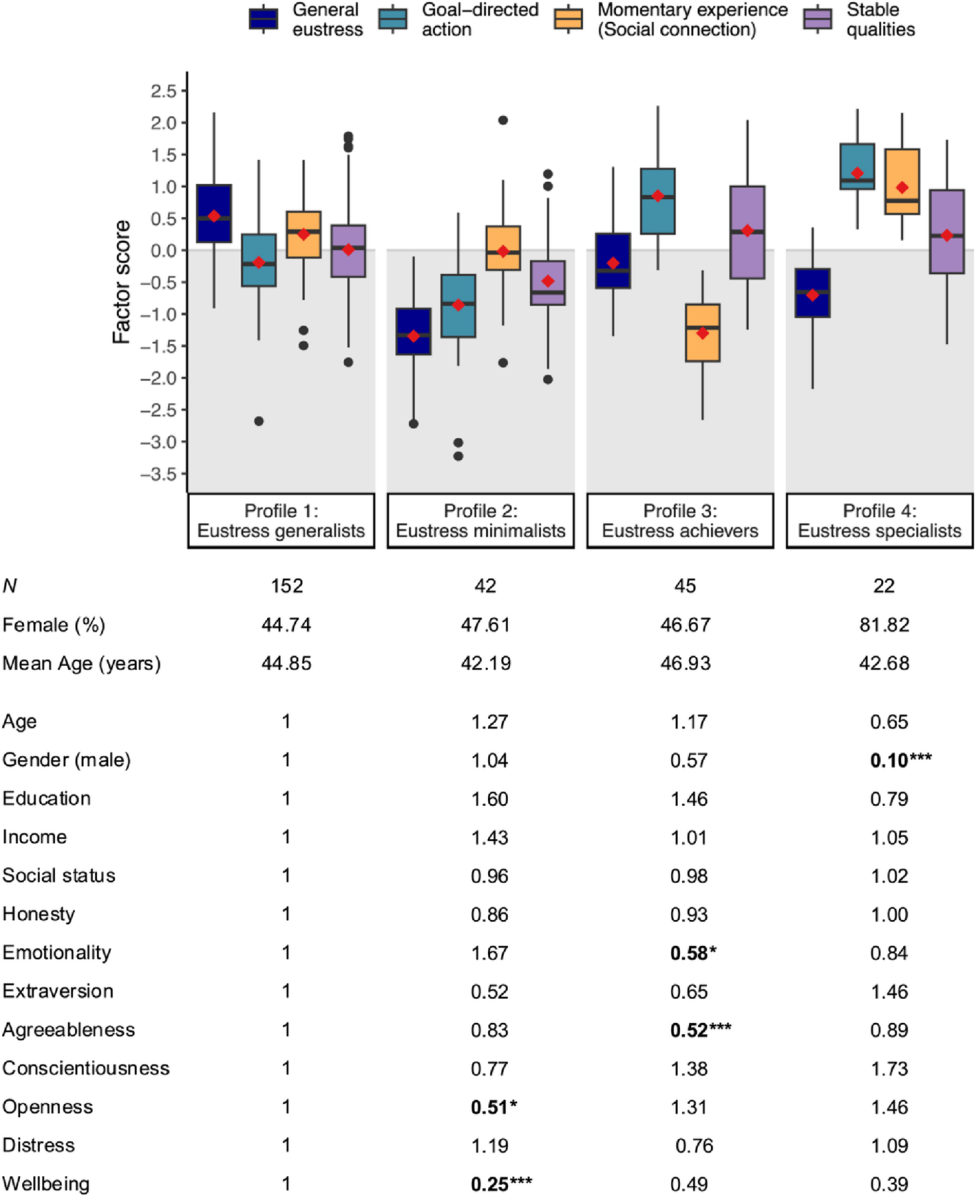
Four identified eustress profiles with odd-ratios for various predictors. *Note*. Boxplots show standardized factor scores of the four indicator variables summarizing data from all participants assigned to the respective profile. Light gray rectangles distinguish negative values below the average score of the full sample (anchored at zero). The lower panel shows the odd ratios for various predictors, indicating the relative probability of being assigned to a specific profile instead of the reference profile 1 (largest profile). The coefficients depict the change of a one-unit increase in the predictor variable (e.g., one standard deviation) or the change from the reference level to the effect level for categorical variables (e.g., from “female” to “male”). *N* = 260 (*n*_*gender*_ = 255). Significant odd ratios are bolded and marked with * *p* <.05; ** *p* <.01; *** *p* <.001

Figure 4 presents the eustress profiles, showing the distributions of factor scores from M3 across participants assigned to each of the four identified clusters. Because factor scores are standardized, a factor score of 0.5 indicates that the score is 0.5 SD above the average score of the entire sample (anchored at zero). Vice versa, a factor score of − 0.5 indicates that the score is 0.5 SD below sample average. Profile 1 represents the largest cluster containing 58% of participants. Participants assigned to this cluster can be described as *“eustress generalists*,*”* because they experience above-average levels of general eustress and about-average levels for all other factors. Profile 2 (16% of participants) represents individuals with below-average eustress scores across all factors. Compared to all other profiles, individuals from profile 2 have the lowest mean scores for three out of four factors—general eustress, goal-directed action, and stable qualities. These indivduals can therefore be construed as *“eustress minimalists”*. Profile 3 (17% of participants) represents “*eustress achievers*,” who experience high levels of eustress while pursuing goals and also internalize eustress as a stable quality but score far below-average on momentary experience items that emphasize social connection. Finally, profile 4 (8% of participants) represents individuals who present with above-average scores of eustress across all the group-level factors but not the general eustress factor. Notably, individuals assigned to this profile experience the highest mean levels of goal-directed action and momentary experience compared to individuals from all other profiles. Individuals from profile 4 may therefore be described as *“eustress specialists”*, thereby contrasting with *“eustress generalists”* from profile 1.

Interestingly, our latent eustress profiles are conceptually similar to profiles from the broaden-and-build theory of positive emotion (Fredrickson & Joiner, 2002). “Eustress generalists” are similar to flourishers who experience high levels of positive momentary engagement and develop positive stable qualities. Vice versa, “eustress minimalists” may reflect languishers who score below average for positive psychological and social functioning and report a lack of resilience when facing difficult situations (Keyes, 2002). Including above-average and below-average scores, “eustress achievers” may be similar to a mixed profile of positive emotion that was established empirically in Winter et al. (2021). Whereas “eustress specialists” may reflect a second mixed eustress profile, the relatively small number of participants fitting this profile (*n* = 22) may also indicate that the model overfitted the data, resulting in a spurious profile (Gerlach et al., 2018). Assessing the validity of this profile in future work would be useful.

#### Individual Differences Between Profiles

We next investigated associations of the four distinct profiles of eustress with sociodemographic characteristics, personality traits, distress, and wellbeing. To do so, we implemented a multinomial logistic regression model that computed odd ratios (*OR*) for all entered predictor variables relative to a reference profile. The odd ratios for the reference profile were anchored at 1. For the other profiles, *OR* > 1 suggests an increased relative probability of the predictor compared to the reference profile, whereas *OR* < 1 suggesst a decreased relative probability. We chose profile 1 as the reference profile because it represented the largest cluster (152 of 260 participants). As predictors, we included five socio-demographic characteristics (age, gender, education, income, and subjective social status; Adler et al., 2000), six personality dimensions (honesty, emotionality, extraversion, agreeableness, conscientiousness, and openness; Ashton & Lee, 2009), distress (PSS-10; Cohen et al., 1983), and psychological wellbeing (ESS Wellbeing; Huppert & So, 2013). We standardized all predictors except gender, which was entered as a binary variable (male vs. female participants; *n*_*gender*_ = 255). The lower panel of Fig. 4 presents the odd ratios with significance levels extracted from Wald z-tests.

Overall, no single predictor discriminated significantly between individuals across all four clusters. We did, however, observe significant differences between profile 1 (reference profile) and the other three profiles for some predictors. Assignment to profile 2 instead of profile 1 was only half as likely for individuals with increased openness (*OR* = 0.51), and only a quarter as likely for individuals with higher psychological wellbeing (*OR* = 0.25). This suggests that *“eustress minimalists”* may be less open to experiencing challenges as something positive, and that they may have fewer positive experiences in life, especially in non-challenging situations (as assessed for wellbeing). Individuals with higher emotionality (*OR* = 0.58) and higher agreeableness (*OR* = 0.52) were only about half as likely to be assigned to profile 3 compared to profile 1. This suggests that these *“eustress achievers*,*”* who generate high eustress from goal achievement but little from social connection, often show lower levels of emotionality and lower levels of agreeableness. Finally, male individuals only had 0.10 odds of being assigned as *“eustress specialists”* (profile 4) compared to *“eustress generalists”* (profile 1). This finding suggests that the males in our sample (compared to females) were much more likely to encounter eustress as a general experience instead of as specific kinds of eustress states.

## Discussion

Here we developed the Comprehensive Hierarchical Eustress Review (CHER), the first psychometric instrument to comprehensively assess positive experiences in challenging situations. CHER includes 47 items, divided into 3 subscales that reflect the 47 low-level features of the CHE model, and implicitly, its 3 general sources of eustress: goal-directed action, momentary experience, and stable qualities of the individual (Kloidt & Barsalou, 2024). Developing this comprehensive instrument allowed us to investigate the psychometric structure of eustress, quantify relationships with related constructs, and establish groups of individuals with distinct eustress profiles.

### RQ1: Unidimensional and Multidimensional Structure of Eustress

In a study with 260 UK participants, a variety of confirmatory factor solutions were examined to establish whether the psychometric structure of eustress is unidimensional, multidimensional, or both. We settled on a bifactor model (M3) as the best-fitting and most conceptually sound solution, indicating that the construct of eustress is weakly multidimensional, consisting of a dominant general factor and several weaker specific (group) factors that reflect CHER’s subscales. Out of 47 candidate items, the best performing bifactor model retained 35 items from all three subscales that captured the eustress construct meaningfully.

Interestingly, items associated with momentary engagement in CHER’s subscale for momentary experience loaded highest on the general eustress factor, together with items for stable qualities (Fig. 2). Thus, experiencing a high level of general eustress was associated with high levels of momentary engagement along with having stable eustress qualities. The group factors captured additional variance in eustress related to goal-directed action, stable qualities, and social connection (the remaining items for momentary experience). Figure 3 captures this overall structure, with 35 items across CHE’s 3 eustress sources forming a tightly integrated core cluster for general eustress, which then differentiates into 3 closely adjoining regions for the 3 sources. Because the remaining 12 candidate items were peripheral to the construct, they were excluded from the model, yielding a more coherent measure of eustress that still reflected its theorized structure.

### RQ2: Eustress, Distress, and Wellbeing

The best performing model, M3, not only exhibited satisfactory internal qualities (i.e., test reliability and discriminatory power), it also exhibited satisfactory external qualities. Specifically, the general eustress factor showed the expected relationships with distress (negative), another eustress scale (positive), and psychological wellbeing (positive). Notably, eustress and distress were only moderately related, indicating that they are not simple inverses of each other. Whereas eustress and distress may share common underlying demands associated with challenging situations, eustress may differ from distress by emphasizing individuals’ coping skills, thereby shifting from threat to challenge appraisals (Lazarus & Folkman, 1984, 1987). Additionally, the largest correlation was between general eustress and wellbeing, suggesting that substantial overlap exists between positive experiences in both challenging situations and non-challenging situations (cf. Kloidt & Barsalou, 2024).

### RQ3: Clusters of Individuals with Different Eustress Profiles

Using the best performing bifactor model, we performed latent profile analysis to establish groups of individuals with different eustress profiles in our sample. We then assessed whether these profiles differed systematically across sociodemographic characteristics, personality traits, distress, and wellbeing. The best-performing solution revealed four profiles that exhibited additional differences across other individual characteristics (e.g., wellbeing, personality traits, gender). Although the extracted eustress profiles conceptually overlapped with typologies for positive emotions (Fredrickson & Joiner, 2002; Keyes, 2002; Winter et al., 2021), future research should assess their robustness across other samples (Gerlach et al., 2018).

### Evaluating and Refining Eustress Theory

Establishing the psychometric structure of eustress offers an empirical assessment of existing eustress theories. Most directly, CHER’s psychometric structure supports the CHE model of eustress in two ways (Kloidt & Barsalou, 2024). First, the presence of a general eustress factor clearly indicates that positive experiences in challenging situations form a coherent construct of eustress. Second, the further presence of multidimensional structure supports CHE’s proposal that the eustress construct also exhibits a multidimensional character across three sources of eustress: goal-directed action, momentary experience, and stable qualities of the individual. The presence of these latter factors confirms the eustress sources identified in CHE, supporting the CHE model (and the literatures on which it is based).

By establishing the features that constitute eustress generally and each of its sources specifically, CHER’s psychometric structure further refines the CHE model. Item loadings in the best-performing bifactor model suggest that the general eustress factor emphasized momentary engagement (i.e., *flourishing/thriving, *and* happiness/joy*) and stable qualities (i.e., *optimistic/positive/cheerful, wellbeing, quality of life*). Additionally, item loadings on the factor for goal-directed action emphasized cognitive processes (i.e., *think/reason effectively, perceived control,* and *insightful appraisals*); item loadings on the factor for momentary experience emphasized social connection (i.e., *compassion/empathy, good relationships,* and *sharing*); and item loadings on the factor for stable qualities factor emphasized personal characteristics (i.e., *self-control, self-esteem,* and *resilience*). Together, these features appear central to constituting positive experiences in challenging situations.

### Improving Eustress Assessments

Determining the psychometric structure of eustress further allowed for an empirical assessment of existing eustress instruments. Most of the previously developed psychometric instruments primarily targeted eustress as the outcome of goal-directed action, failing to measure eustress as an outcome of momentary experience or stable qualities (Cavanaugh et al., 2000; O’Sullivan, 2011; Rodríguez et al., 2013). Assessing CHE’s 47 eustress features, however, revealed that items from all sources of eustress reside at the center of the construct. Perhaps most notably, the strongest factor loadings on the general factor were for items from the subscales for momentary experience and stable qualities (*not* from goal-directed action). Ignoring these sources of eustress in psychometric assessment may therefore lead to incomplete or even misleading assessment. Thorough assessments of eustress instead are likely to benefit from a comprehensive set of items, such as the one in CHER that follows from the CHE model.

The results reported here from assessing CHER in a UK-adult sample offer a potential basis for subscales and items to be included in future eustress assessments. Sampling strongly loading items from the general factor could allow for a straightforward assessment of eustress, similar to general assessments of distress (e.g., PSS-10; Cohen et al., 1983). A general eustress scale also seems useful for capturing important links between eustress, distress, and wellbeing. Conversely, sampling strongly loading items from the specific factors provides the opportunity to assess individual differences and structural relations between the three eustress sources. A multidimensional eustress scale also seems useful for establishing nuanced eustress profiles in a population. Future research could therefore profitably develop and evaluate short versions of CHER that serve a wide variety of research purposes.

### Boosting Eustress Experiences

In addition to the theoretical and empirical contributions that the study here makes, it also has potential implications to inform practical applications. For example, we identified four different clusters of individuals who exhibited varying levels of eustress levels, along with correlated patterns of individual difference measures. By using these eustress profiles to inform the selection of interventions, optimal interventions could be tailored to maximizing eustress in particular segments of the population. For example, one eustress profile exhibited a high level of eustress for goal-achievement but not for momentary experience, neither as social connection nor as momentary engagement (“*eustress achievers*”). For these individuals, a focus on developing skills associated with social connection and momentary engagement might be most effective for increasing their overall eustress levels.

More generally, when an individual exhibits a low score for one or more of CHER’s subscales, they could receive training to increase the respective source(s) of eustress. For example, eustress that results from goal-directed action could be boosted effectively by training skills that foster successful goal achievement and problem solving, whereas eustress that results from momentary experience could be boosted by strengthening skills associated with savoring and mindfulness, or by strengthening prosocial skills and social connection. Finally, eustress that results from stable qualities could be strengthened by working to establish new eustress habits.

 Ultimately, interventions that boost eustress may have desirable spillover effects. As we saw in Table 2, higher levels of general eustress were correlated with decreased distress and increased wellbeing. To examine these relationships more carefully, future research could assess whether increasing eustress causally decreases distress (i.e., eustress as an antidote to distress) and causally increases wellbeing (i.e., eustress as an ally to wellbeing). Also of interest is whether increasing wellbeing across all situations causally increases eustress in challenging situations (Kloidt & Barsalou, 2024).

### Towards Robust Population-Level Inferences

The inferential power of the analyses and results for this project are limited by its non-probabilistic sample design, which does not provide information about the selection probabilities from the adult UK population (Heeringa et al., 2017). Although the basic demographics of our sample approximated adult UK population values, we cannot automatically assume that the results from our measurement models will generalize to other samples or to the general population. Although this project develops a novel eustress instrument that is appropriate for assessing eustress in the general population, the initial evaluation presented here provides insights into a specific sample of UK adults. To establish robust population-level inferences, we therefore invite future research to replicate and extend our analyses with probabilistic sampling methods.

## Conclusion

The Comprehensive Hierarchical Eustress Review (CHER) developed and presented here is the first psychometric instrument to comprehensively assess positive experiences in challenging situations, while providing psychometric evidence of its quality. Determining the psychometric structure of eustress as measured with CHER further supported and refined the CHE model of eustress. Quantifying relationships with related constructs highlighted how promoting eustress may be related to increased wellbeing and decreased distress. The results with CHER here also offer an initial basis for measuring general eustress comprehensively in an adult UK sample, along with its three specific sources of goal-directed action, momentary experience, and stable qualities. Finally, identifying clusters of individuals with distinct eustress profiles can support the design of specific eustress interventions for different kinds of individuals.

## Appendix

**Fig. 5:**
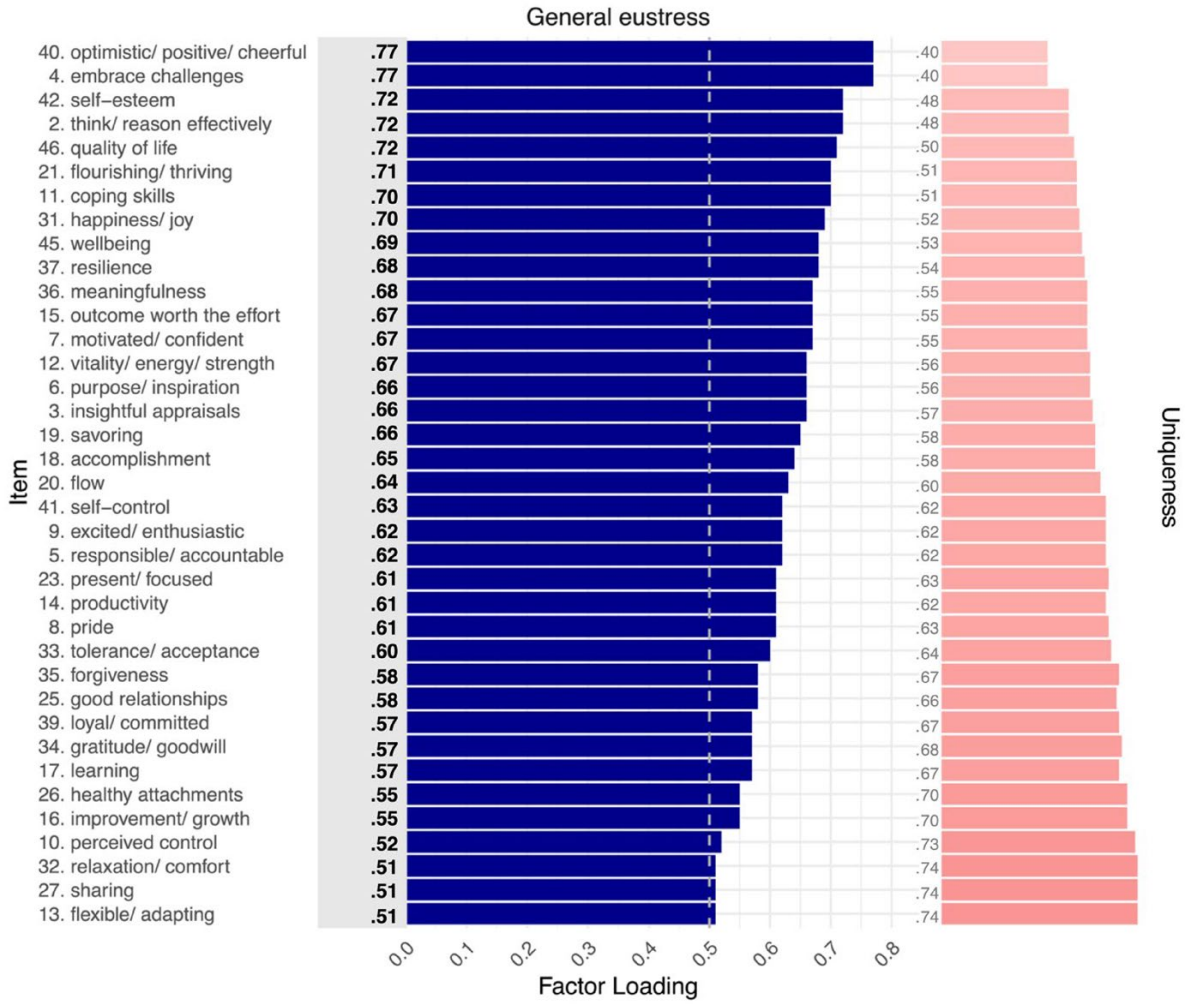
Factor loading plot for Model 1 (unidimensional). *Note*. M1 captured 37 (out of 47) CHER items with main factor loadings ≥ 0.50 (dashed gray line). M1 accounted for 40.41% of total variance. Factor loadings ≥ 0.50 are bolded. Light gray rectangles distinguish negative factor loadings. *N* = 260

**Fig. 6:**
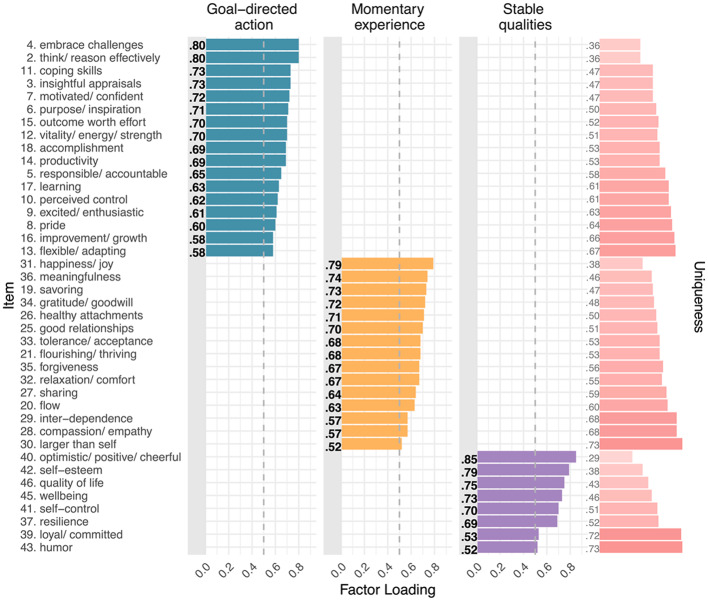
Factor loading plot for Model 2 (correlated factors). *Note*. M2 captured 40 (out of 47) CHER items with main factor loadings ≥ 0.50 (dashed gray line). The correlated factors modelled CHER’s subscales of goal directed action (17 items), momentary experience (15 items), and stable qualities (8 items). M3 accounted for 46.54% of total variance, with most variance explained by goal-directed action (42.48%), followed by momentary experience (36.27%), and stable qualities (21.25%). Factor loadings ≥ 0.50 are bolded. Light gray rectangles distinguish negative factor loadings. *N* = 260

See Figs. 5, 6 and Table 3.

**Table 3:**
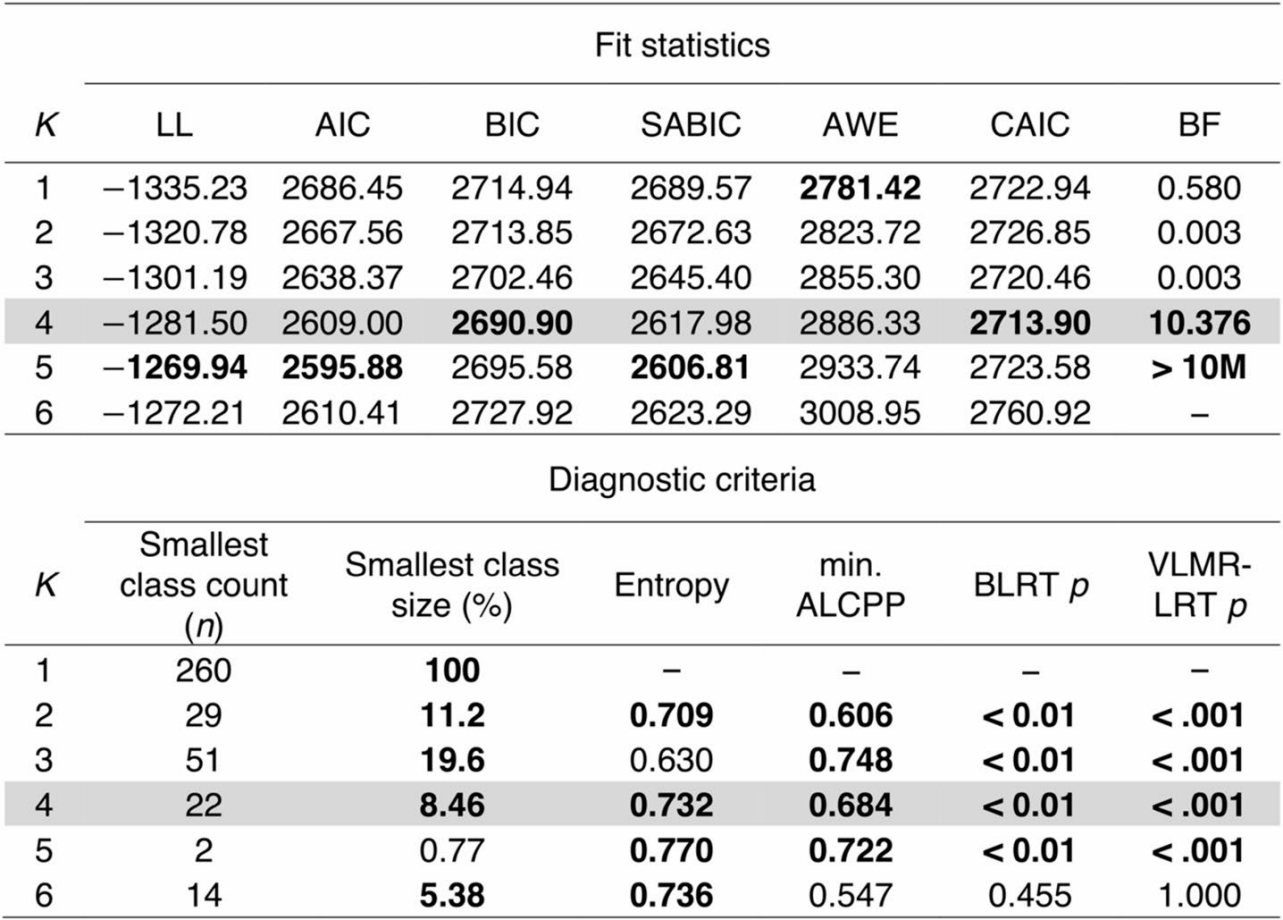
Latent profile analysis models: Fit statistics and diagnostic criteria

*Note*. Bolded values indicate satisfactory fit for each respective statistic. The 5-cluster solution had convergence issues and was therefore dismissed for model selection. The chosen solution is shaded in gray. *K* = number of clusters; LL = log-likelihood; AIC = Akaike information criterion; BIC = Bayesian information criterion; SABIC = sample-size adjusted BIC; AWE = approximate weight of evidence criterion; CAIC = consistent AIC; BF = Bayes factor; min. ALCPP = minimum of the average latent class posterior probability; BLRT = bootstrapped likelihood ratio test; p = p-value; VLMR-LRT = Vuong-Lo- Mendell-Rubin adjusted likelihood ratio test. *N* = 260

## Data Availability

Data files and analysis scripts are publicly accessible on OSF at https://osf.io/s7mgy/. All other materials used in the research are included in the manuscript and supplemental materials.
